# Histone deacetylase inhibition leads to neuroprotection through regulation on glial function

**DOI:** 10.1186/1750-1326-8-S1-P49

**Published:** 2013-09-13

**Authors:** Xuefei Wu, Shao Li, Qiong Wu, Yan Peng, Deqin Yu, Hecheng Wang, Dehua Chui, Jie Zhao

**Affiliations:** 1 Department of Physiology, Dalian, Liaoning Province, China; 2 Neuroscience Research Institute, Peking University, China

## Background

Epigenetic mechanisms such as post-translational histone acetylation are increasingly recognized for their contribution to gene activation and silencing in the brain and contribute to neurodegeneration. Acetylation degree of histones is highly regulated by the enzymes known as histone acetyltransferases (HATs) or histone deacetylases (HDACs). HDAC inhibition affects the expression of only a small subset of genes, leading to transcriptional activation or repression through hyperacetylation of histone or non-histone proteins. HDAC inhibitors are a class of compounds that interfere with the function of HDAC and have been viewed as promising agents to combat neurodegenerative diseases such as Alzheimer’s disease (AD) and Parkinson’s disease (PD). Multiple mechanisms underlying the effects of HDAC inhibitors on neuroprotection and restoration of memory and motor impairments in AD or PD models have been proposed, however, little is known about the contribution of HDAC inhibition in glial cells. It has been reported that valproic acid (VPA), a drug commonly used for epilepsy and bipolar disorders, upregulates the expression of neurotrophic factors, including glial cell line-derived neurotrophic factor (GDNF) and brain-derived neurotrophic factor (BDNF) in astrocytes, which contributes to VPA-mediated neurotrophic effect on dopamine neurons. VPA also induces microglia apoptosis and suppresses the production of proinflammatory factors in microglia. As VPA is a HDAC inhibitor, we hypothesize that HDAC inhibition may contribute to neuroprotection through regulating gene expressions in glial cells.

## Materials and methods

We thus investigated the effects of several HDAC inhibitors including VPA, trichostatin (TSA), sodium phenylbutyrate (4-PBA) and nicotinamide, on neurotrophic and proinflammatory functions of astrocytes and microglia.

## Results

Our results indicate that (1) VPA and TSA increased GDNF and BDNF transcripts in astrocytes that might be attributable, at least in part, to histone hyperacetylation in specific gene promoters as GDNF promoter activity and promoter-associated histone H3 acetylation level were elevated; (2) both 4-PBA and nicotinamide decreased the cell viability and release of TNF alpha and nitric oxide from cultured microglia stimulated with the inflammagen lipopolysaccharide (LPS). (3) VPA, TSA and 4-PBA protected dopamine neurons in neuron/glia co-cultures challenged with MPTP or LPS; (4) PBA reduced TNF alpha expression in the brain of C57 mice intraperitoneally injected with LPS with no effect on microglia cell count.

## Conclusions

In summary, histone acetylation is involved in regulation of glial functions and may thus contributes to neurodegneration or neuropretection. Further studies on HDAC inhibition in glial cells in AD and PD animal models are warranted to gain a more comprehensive understanding of the mechanisms underlying actions of HDAC inhibitors in the brain.

